# Downscaling Pest Risk Analyses: Identifying Current and Future Potentially Suitable Habitats for *Parthenium hysterophorus* with Particular Reference to Europe and North Africa

**DOI:** 10.1371/journal.pone.0132807

**Published:** 2015-09-01

**Authors:** Darren J. Kriticos, Sarah Brunel, Noboru Ota, Guillaume Fried, Alfons G. J. M. Oude Lansink, F. Dane Panetta, T. V. Ramachandra Prasad, Asad Shabbir, Tuvia Yaacoby

**Affiliations:** 1 CSIRO, GPO Box 1700, Canberra, ACT, Australia; 2 European and Mediterranean Plant Protection Organization, Paris, France; 3 CSIRO, Private Bag 5, Wembley, WA, Australia; 4 Anses, Laboratoire de la Santé des Végétaux, Montferrier-sur-Lez cedex, France; 5 Wageningen University, PO Box 8130, Wageningen, The Netherlands; 6 The University of Melbourne, Parkville, VIC, Australia; 7 Directorate of Weed Science Research Centre, University of Agricultural Sciences, Bengaluru, India; 8 Department of Botany, University of the Punjab, Lahore, Pakistan; 9 Plant Protection and Inspection Services, Bet Dagan Agricultural Center, Ministry of Agriculture and Rural Development, P.O. Box 78, Bet-Dagan, Israel; DOE Pacific Northwest National Laboratory, UNITED STATES

## Abstract

Pest Risk Assessments (PRAs) routinely employ climatic niche models to identify *endangered* areas. Typically, these models consider only climatic factors, ignoring the ‘Swiss Cheese’ nature of species ranges due to the interplay of climatic and habitat factors. As part of a PRA conducted for the European and Mediterranean Plant Protection Organization, we developed a climatic niche model for *Parthenium hysterophorus*, explicitly including the effects of irrigation where it was known to be practiced. We then downscaled the climatic risk model using two different methods to identify the suitable habitat types: expert opinion (following the EPPO PRA guidelines) and inferred from the global spatial distribution. The PRA revealed a substantial risk to the EPPO region and Central and Western Africa, highlighting the desirability of avoiding an invasion by *P*. *hysterophorus*. We also consider the effects of climate change on the modelled risks. The climate change scenario indicated the risk of substantial further spread of *P*. *hysterophorus* in temperate northern hemisphere regions (North America, Europe and the northern Middle East), and also high elevation equatorial regions (Western Brazil, Central Africa, and South East Asia) if minimum temperatures increase substantially. Downscaling the climate model using habitat factors resulted in substantial (approximately 22–53%) reductions in the areas estimated to be endangered. Applying expert assessments as to suitable habitat classes resulted in the greatest reduction in the estimated endangered area, whereas inferring suitable habitats factors from distribution data identified more land use classes and a larger endangered area. Despite some scaling issues with using a globally conformal Land Use Systems dataset, the inferential downscaling method shows promise as a routine addition to the PRA toolkit, as either a direct model component, or simply as a means of better informing an expert assessment of the suitable habitat types.

## Introduction

Whilst the roots of pest risk modelling extend back to early in the 20^th^ Century [1], modern computer-based pest risk modelling has only been practised for some 30 years [2,3]. In that time, there has been a progressive refinement of the spatial distributions of the modelled risks. In the earliest maps, risks were portrayed wherever climate stations were situated [2]. Following the development of climatic splining techniques [4], spatially interpolated results were presented e.g., [5,6]. Increased computing power, and a thirst for more detailed risk maps saw the development of finer-scaled gridded climate datasets [7,8,9], and their application to pest risk modelling problems e.g., [10,11,12].

Under the International Standards for Phytosanitary Measures (ISPM’s), Pest Risk Assessments (PRAs) need to identify the *endangered area*, “an area where ecological factors favour the establishment of a pest whose presence in the area will result in economically important loss” [13]. Whilst the standards define the *area* as “…an officially defined country, part of a country, or all or part of several countries”, the Decision-support scheme for quarantine pests of the European and Mediterranean Plant Protection Organisation [14] encourages the risk assessor to define the endangered area at a very fine ecological and geographical scale. In order to achieve this, it is not sufficient to use even finer resolution climate datasets. Ecological theory indicates that we need to consider the effects of non-climatic factors as we investigate species niches at finer geographical scales [15].

Considering the non-climatic factors affecting a species potential distribution can be a challenging prospect. Many factors could affect the potential habitat suitability for a species, and the importance and effect of these factors may often, themselves, depend on climatic factors [1,16,17]. For example, topographic features that concentrate overland flow of water may improve the suitability of habitat at the dry end of the species' potential range, helping it to avoid drought stress; conversely, at the wet end of the range, this same factor may decrease habitat suitability due to waterlogging. Whilst it is theoretically possible for correlative species distribution models to uncover such relationships, the inclusion of these variables in models may add further to the notorious problems of model over-fitting. This will have the effect of diminishing model transferability; consequently reducing even further the value of such models for pre-border pest risk applications.

Until ecological niche modelling methods improve to the point where these non-climatic factors can be better understood and incorporated into modelling frameworks appropriately, there is a need for a practical risk analysis method that can refine a climatic analysis. Baker *et al*. [18] is amongst the earliest attempts to incorporate non-climatic information into a PRA, combining a CLIMEX model of climate suitability with a crop host distribution map for *Diabrotica virgifera virgifera*. In order to assess the pest risks from invasive alien species more precisely, one prospect is to extend the method of Baker *et al*. [18], combining the semi-mechanistic climate modelling methods with spatial land use. In the present study, we use *Parthenium hysterophorus* (Asteraceae) as a case study.

*Parthenium hysterophorus* is an annual or short-lived perennial plant native to the subtropics of North and South America. It is a notorious invasive species which has spread to Australia, Africa, Asia, Oceania, and the Middle-east, where it has become a serious agricultural and rangeland weed affecting crop production and animal husbandry, as well as human health and biodiversity [19,20].

Within the European and Mediterranean Plant Protection Organization region, *P*. *hysterophorus* is presently officially recorded only in Israel [21]. It is recorded as naturalised in Egypt [22] and it has also been observed as casual in Belgium [23] and Poland [24]. It is thought to have been introduced in Israel in 1980, probably through the import of contaminated grains from the USA for use as fish food in ponds [25]. The species was also introduced in India and Ethiopia, possibly as a contaminant of grain from the USA. In addition, there are records of its introduction as a contaminant of pasture seed and food aid [26], and through the movement of animals and seed attached to used vehicles (harvesters, military machinery, and other vehicles) [27].

*Parthenium hysterophorus* reproduces by seeds and is known to be highly prolific, as a single plant may produce on average 40 000 seeds [28]. These seeds are dispersed locally by wind and water and as a contaminant of hay, seed, harvested material, soil, vehicles, machinery, or animals. *Parthenium hysterophorus* seeds exhibit dormancy mechanisms and can form persistent seed banks, especially where the seeds are incorporated into soil at moderate depths [29]. The species tolerates a wide variety of soils and is a pioneer that can colonise a wide range of habitats: grazing land, summer crops, disturbed and cultivated areas, roadsides, recreation areas, as well as riverbanks and floodplains. *Parthenium hysterophorus* matures very quickly, with flowering commencing 4–6 weeks after germination; given suitable temperatures it can establish in areas receiving very low rainfall [30].

*Parthenium hysterophorus* causes major negative impacts on pastures and crops. In India, it has been observed that *P*. *hysterophorus* can cause yield losses of up to 40% in several dryland crops [31] cited in [32]. In Ethiopia, the yield of *Sorghum bicolor* grain was reduced by between 40 and 97% when *P*. *hysterophorus* was left uncontrolled throughout the growing season [33]. In Queensland (Australia), it has invaded 170 000 km² of high quality grazing areas and losses to the cattle industry have been estimated to be AUD$22 million per year in control costs and loss of pasture [34]. Infestations of *P*. *hysterophorus* can also degrade natural ecosystems, and outcompete native plant species [35,36]. Because *P*. *hysterophorus* contains sesquiterpenes and phenolics, it is toxic to cattle, horses and other animals [30]. In addition, meat and milk produced from livestock that has eaten the plant can develop an undesirable flavour [37]. Frequent contact with *P*. *hysterophorus* or its pollen can produce serious allergic reactions such as dermatitis, hay fever and asthma in humans and livestock, especially horses [38].

The impacts of *P*. *hysterophorus* and reports of its presence in Israel and Belgium sparked concern within the EPPO region and a desire for a PRA to gauge the extent of the threat it posed [39]. A critical component of pest risk is an understanding of the potential distribution of the pest within the PRA area. McConnachie *et al*. [40] presents a CLIMEX model of *P*. *hysterophorus* based on its then known distribution and experimental observations drawn from the scientific literature. In the light of the present known distribution of *P*. *hysterophorus*, the CLIMEX model of McConnachie *et al*. appears somewhat conservative, especially with respect to the cold tolerance limits of this species.

In this paper we refit the CLIMEX model of *P*. *hysterophorus* developed by McConnachie *et al*. [40], and apply irrigation and climate change scenarios to inform global pest risks. We extend the methods of Baker *et al*. [18] using readily available habitat data, comparing two methods for downscaling the risk map, globally, and for Europe. The first method uses the standard EPPO PRA procedure involving expert assessment of the habitat types that are suitable for invasion, while the second uses an objective inferential method.

## Materials and Methods

### Modelling outline

**Fig 1:**
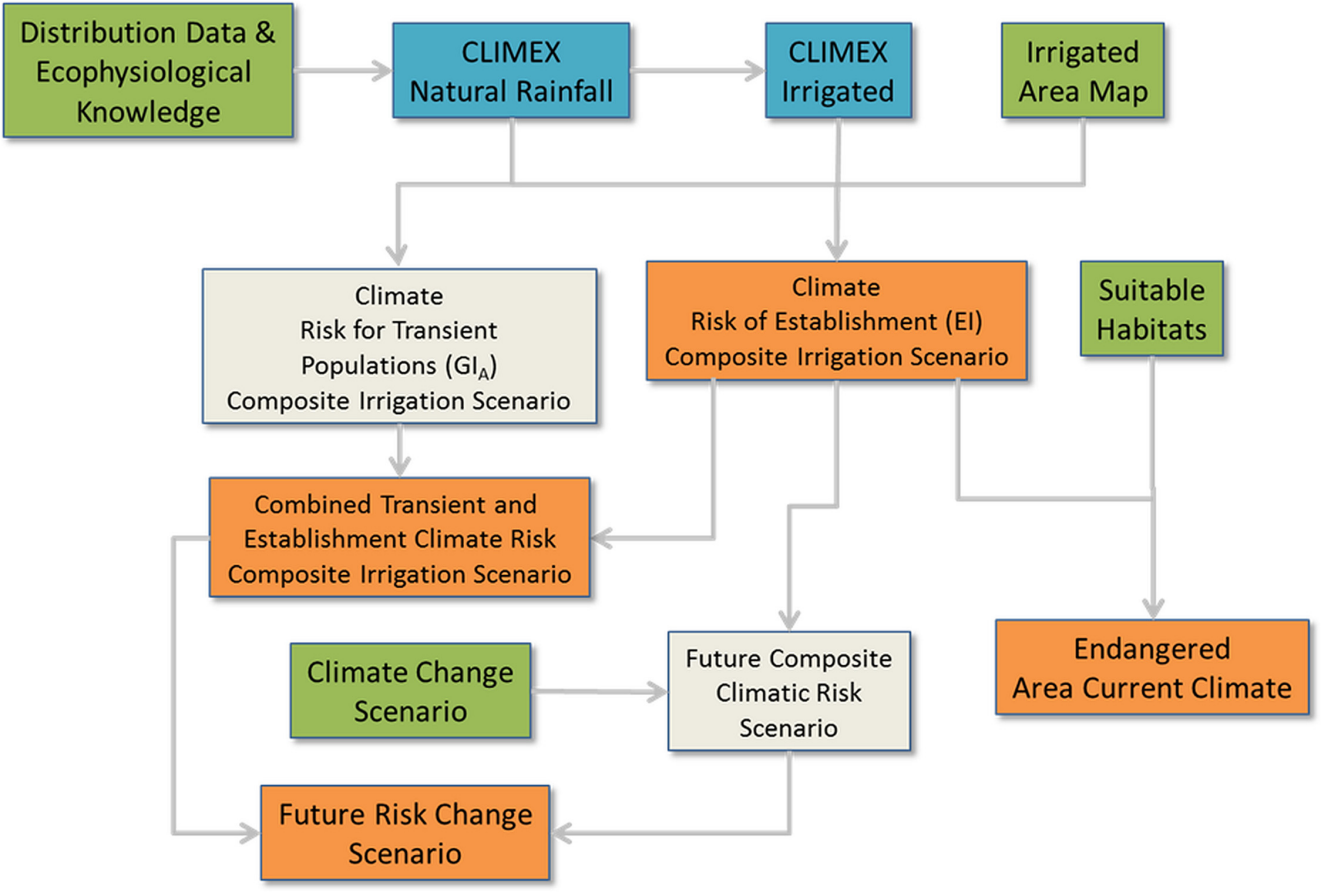
Modelling scheme for assessing pest risks for *Parthenium hysterophorus* in the EPPO region using the EPPO Decision-support scheme for quarantine pests. Green boxes are inputs, blue boxes are models, grey is an intermediate product, and orange boxes are outputs.

The modelling scheme is presented in Fig 1. The distribution data and ecophysiological knowledge for *P*. *hysterophorus* were used to develop a CLIMEX model under natural rainfall conditions. Because some distribution records for *P*. *hysterophorus* appear to represent populations that are able to persist only due to the presence of supplementary soil moisture, the CLIMEX model is used to run a natural rainfall and an irrigation scenario. These model outputs are combined on a cell-by-cell basis using a map of the distribution of irrigation areas [41] to create composite climate risk models for transient and established populations. The suitable habitat types are used to refine the climate suitability map for establishment to create the endangered area map for the risk assessment. A climate change scenario based on a Global Climate Model is then used to create a future composite climate risk scenario as a means of better understanding the sensitivity of any policy responses to the risks posed by *P*. *hysterophorus*.

### Distribution data

**Fig 2:**
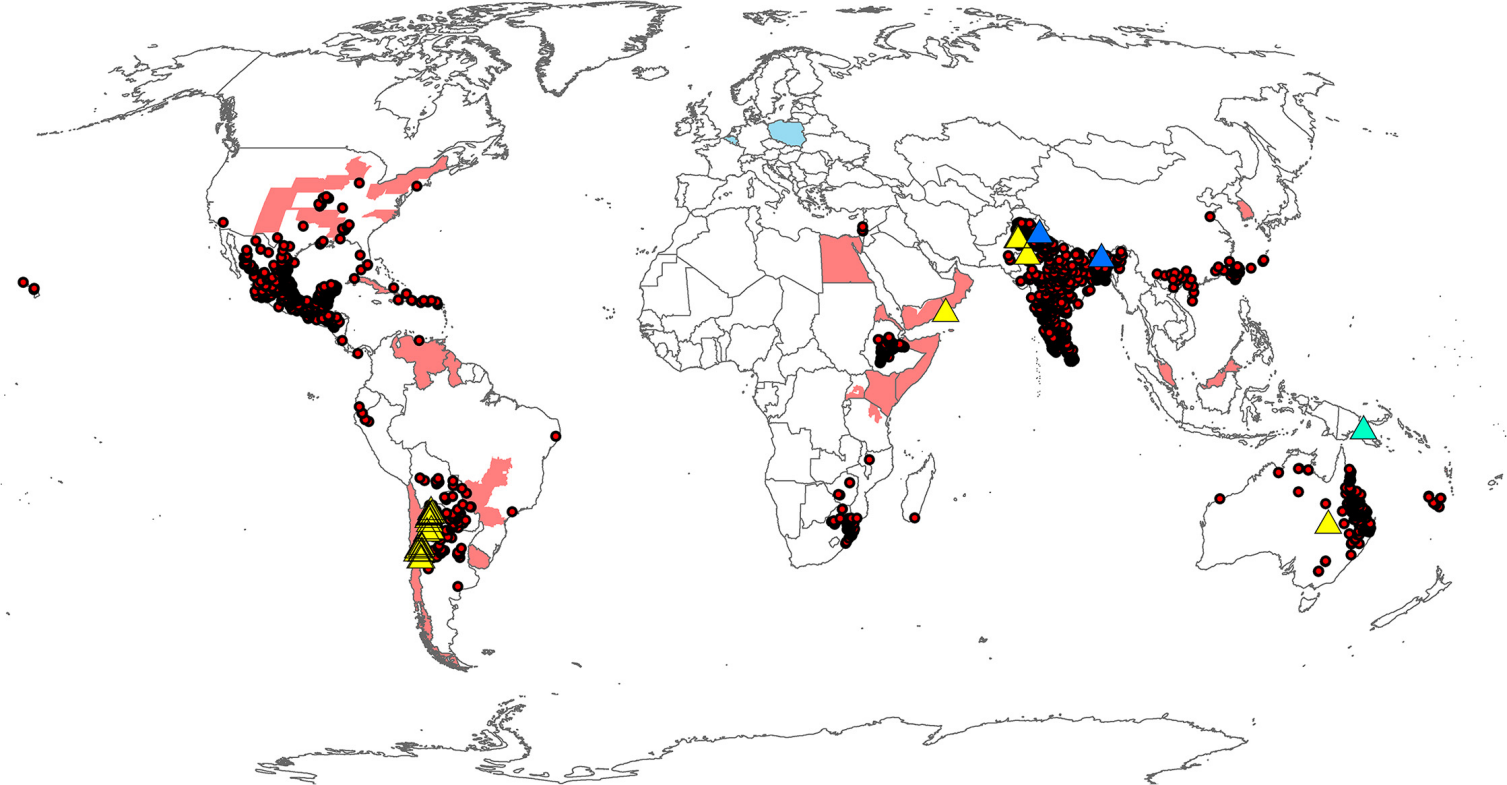
Known global distribution of *Parthenium hysterophorus*. Red circles represent distribution points where *P*. *hysterophorus* is known to be established, blue triangles indicate outliers in apparently excessively cold locations, yellow triangles excessively dry locations, green triangles excessively wet locations. Pink areas represent national or sub-national administrative units where the species has been recorded established, blue areas indicate countries where the species has been reported as transient populations.

The known distribution of *P*. *hysterophorus* was assembled from the Global Biodiversity Information Facility (www.gbif.org), Clark & Lotter [42], Dhileepan [43], Department of Natural Resources [44], Kilian *et al*. [45], and Shabbir *et al*. [46] (Fig 2). Administrative regions that had been reported as being infested by *P*. *hysterophorus*, but had no point location records were added to the distribution map as polygons, and shaded lightly to reinforce the lack of spatial precision of these reports. The 2 536 point distribution records were transformed into shapefiles and imported into CLIMEX for overlaying results during model fitting. During model fitting for the natural rainfall scenario, records were checked to consider whether the populations were likely to be able to persist in the absence of irrigation, and whether they represented *Established* or *Transient* populations (*sensu* FAO [13]).

### CLIMEX modelling

CLIMEX V3 [2,47] was used to refit the model of McConnachie *et al*. [40] for *P*. *hysterophorus*. CLIMEX calculates a weekly Growth Index (GI_W_) that describes the species population response to temperature and soil moisture through the Temperature (TI) and Soil Moisture (MI) indices respectively. GI_W_ is integrated annually to calculate the Annual Growth Index (GI_A_). Stress indices (hot, cold, wet, dry) are factors that limit a species’ ability to persist at a particular location. Individual stress values are combined to create the total Stress Index (SI), and when combined with the Annual Growth Index (GI_A_) CLIMEX calculates the Ecoclimatic index (EI). The EI is a measure of the overall suitability of a location for species persistence year-round (the larger the value the more suitable). We classified the invasion risk as *Endangered* if the model indicated that *P*. *hysterophorus* was likely to be able to persist year-round (EI>0). At locations where it could grow during a favourable season, but is unlikely to persist year-round due to an inability to complete a generation, due either to stresses or an insufficient heat sum to complete reproductive development (EI = 0, GI_A_>0), we classified it as *Transient* [13] (which is synonymous with casual populations *sensu* Richardson *et al*. [48]).

The model-fitting strategy involved fitting the stresses to the distribution data in the native range in South America, and the introduced range in Africa, India, and North America. Distribution data in Australia and Eastern Asia were reserved for model validation. In fitting the stress and growth functions, consideration was given to any reported experimental data or theoretical expectations. This practice, combined with the structure of the CLIMEX Compare Locations model helps guard against over-fitting [49]. All CLIMEX model parameters for *P*. *hysterophorus* are provided in Table 1, and their derivation is detailed below.

**Table 1:** CLIMEX model parameters for *Parthenium hysterophorus*. Parameter mnemonics follow Sutherst *et al*. [47].

Parameter	Description	*Values*	*Units^†^*
**Moisture**			
SM0	Lower soil moisture threshold	0.1	
SM1	Lower optimal soil moisture	0.3	
SM2	Upper optimal soil moisture	0.8	
SM3	Upper soil moisture threshold	**1.5**	
**Temperature**			
DV0	Lower temperature threshold	6	°C
DV1	Lower optimal temperature	22	°C
DV2	Upper optimal temperature	32	°C
DV3	Upper temperature threshold	39	°C
**Cold stress**			
TTCS	Cold stress temperature threshold	**-7.5**	°C
THCS	Cold stress accumulation rate	**-0.01**	Week^-1^
**Heat stress**			
TTHS	Heat stress temperature threshold	**40**	°C
THHS	Heat stress accumulation rate	0.001	Week^-1^
**Dry stress**			
SMSD	Soil moisture dry stress threshold	0.10	
HDS	Dry stress accumulation rate	**-0.015**	Week^-1^
**Threshold Annual Heat Sum**			
PDD	Annual heat sum threshold	2 000	°C days

^**†**^Units without symbols are a dimensionless index of available soil moisture, scaled from 0 (oven dry), with 1 representing field capacity.

Values in bold face type have been changed from values included in McConnachie et al. [40]

#### Temperature index

Williams and Groves [50] found an optimal temperature regime for P. hysterophorus of 25°C night/30°C day. The Temperature Index parameter values remain unchanged from McConnachie et al. [40].

#### Cold stress

The cold stress threshold and rate parameters of McConnachie et al. [40] were relaxed to allow P. hysterophorus to persist in the known, northern locations in the USA and northern India. In doing so, the extreme cold records in China and northern Pakistan and India also became suitable. Williams and Groves [50] (p. 50) noted that plants that were frosted at -6°C suffered “…leaf damage, leading to complete senescence and lateral floret development ceased”. Using -7.5°C as a damaging cold stress threshold (TTCS), the stress accumulation rate of -0.01 week-1 fitted all bar two of the coldest locality records in the northern hemisphere. The outlying records in the Himalayas are found in a region of extremely dissected topography, and the altitude and temperature are so extremely different to the next closest location records that this is likely to be a case of mismatch in either geocoding precision or the climate data. In Argentina, a number of location records for P. hysterophorus in the GBIF database referred to locations that were apparently too cold or too dry for persistence, and for the dry records, did not appear to fall in irrigation areas defined in the irrigation areas database of Siebert et al. [41]. Searching Google Earth using the locality description of these records revealed that they were incorrectly geocoded, and actually referred to wetter locations found at lower elevations.

#### Dry stress

In the CLIMEX framework, dry stress may not be a factor that affects annual plants directly, because these plants may be able to survive extended periods of drought in the seed life stage. In this case, Dry Stress (in concert with the GIA) acts in such a manner as to ensure that there is a sufficient period within which the soil moisture is sufficient to complete the life cycle. The dry stress accumulation rate was increased to make the westernmost record in Queensland, Australia barely climatically unsuitable. This had the consequence of making some of the records in Pakistan and Western Argentina unsuitable in the absence of irrigation, which was practised there according to the GMIA database of Portmann et al. [51]. In a small number of cases, location records in Argentina (17), Australia (1), India (1) and Pakistan (2) fell in areas that, according to the climate database were extremely xeric and which were not associated with widespread crop irrigation, at least as portrayed in the global irrigated area database we used (see Composite Risk Mapping below). Examining these locations in Google Earth revealed that these records were not able to be related logically to a long-term climatology. The Argentinian records fell in towns or roadsides where there was irrigation or a concentration of rainfall respectively within areas that were extremely sparsely vegetated. The Australian record was within a braided river channel that floods very infrequently due to rain mostly falling further up the catchment. The Indian record fell in Bikaner, a moderately large town that is in the middle of a desert. Bikaner and its surrounding cropping plots are sustained by the Ganges and Indira Ghandi Canals. The Pakistani records were located along a road through an area between the Indus and Chenab Rivers. This area is a desert, which is covered in extremely sparse vegetation, except for some scattered cropping plots.

#### Wet stress

In the native range of P. hysterophorus in South America, there is an extremely large area around the Amazon Basin where the CLIMEX model indicates potential for growth and persistence, but where there are no location records. Whilst this may be due to a lack of surveying and reporting effort, we explored the possibility that P. hysterophorus is unable to persist there due to excessive cloudiness associated with high rainfall (the species is reportedly sensitive to shading [50]. It was possible to make this wet habitat unsuitable using wet stress, improving the model specificity in this area. However, when this level of wet stress was applied, all of Bangladesh, North-eastern India and parts of Central Kenya also became unsuitable; but these areas are covered in location records for P. hysterophorus (see [52] for detailed maps of P. hysterophorus in East Africa. This paradox can perhaps be explained by the fact that whilst the natural vegetation of Bangladesh, North-eastern India, and Central Kenya are similar in structure to that of the Amazon Basin, most of the vegetation in these introduced range locations has been disturbed by intensive agriculture [53]. In the absence of agricultural or pastoral disturbance regimes, we might expect that P. hysterophorus would tend to be outcompeted by the natural vegetation.

#### Annual heat sum threshold

The annual heat sum threshold (PDD) of McConnachie et al. [40] was retained at 2 000°C days above 6°C (DV0), barely allowing P. hysterophorus to persist at the coldest known locations of P. hysterophorus in the Himalaya Mountains.

### Climate data

The model was fitted initially using the 30’ CliMond CM30_1975H_WO_V1.1 dataset, and subsequently refined with the CM10_1975H_WO_V1.1 [9]. The CliMond 10’ results for 2070 of the A2 SRES climate change scenario run on the CSIRO Mk 3 GCM (CM10_2070_CS_A2_WO_V1.1) was chosen because it represented a reasonably extreme scenario that would highlight the sensitivity of the invasion potential for *P*. *hysterophorus*.

### Irrigation

An irrigation scenario of 2.5 mm day^-1^ was applied as a top-up to natural rainfall. Under this scenario, in any week in which average daily precipitation did not meet this threshold, the difference was assumed to be added to the rainfall inputs to the soil moisture model. Actual irrigation rates depend on a variety of factors, including the crops, their stage of growth and climatic factors such as wind flux, temperature, and humidity. The selected rate accords with indicative low-end rates [54]. The irrigation scenario was run on the global CM10_1975H_WO_V1.1 dataset.

### Composite soil moisture risk mapping

The irrigation area map from Siebert *et al*. [41] was used to select within each climate cell, which of the natural and irrigated CLIMEX model results to use in a composite risk map. For each 10’ cell, if the irrigation area was greater than 0, the irrigation scenario results were included. Otherwise the natural rainfall scenario value was used.

### Habitat factors

We compared two methods for identifying habitat types that are suitable for invasion by *P*. *hysterophorus*. The first, loosely termed an expert assessment, reflects the current standard practice within the EPPO pest risk assessment framework, while the second is an objective inferential method.

In the expert assessment, the habitat types listed in the CORINE database [55] were considered by the EPPO Expert Working Group while performing the PRA for *P*. *hysterophorus*, and classified as either suitable or unsuitable for *P*. *hysterophorus* based upon consideration of the habitat types where it has been reported in the literature, and where the panel members had observed it in the field. The CORINE database was selected because it is preferred by the EPPO due to its fine spatial resolution. Notably, the spatial coverage of the CORINE database is limited to Europe. The assessors used a consensus method to decide on suitable land use factors, drawing upon published descriptions and personal observations of *P*. *hysterophorus* occupying different habitat types.

In the inferential method, the distribution points in Fig 2 were spatially intersected with a global habitat dataset; habitat types with one or more point records were listed. This list was used to identify the subset of habitat types in Europe that was considered suitable. Because the geographical coverage of the CORINE database is limited to Europe, the FAO Land Use Systems of the World version 1.1 [www.fao.org/nr/lada/] was used to identify suitable habitat types. This database has a moderately coarse spatial resolution (5 arc minutes) which is equivalent to a map scale of approximately 1:10 000 000. This is coarser than the CORINE database, which summarises the spatial data at a scale of 1:100 000 (equivalent to a raster resolution of approximately 50 m). The attraction of the FAO dataset is that it has a global coverage, enabling risks to be projected globally.

For both the CORINE and FAO datasets, the suitable habitat classes were spatially intersected with the CLIMEX model of climate suitability to create composite climate and land use/habitat risk maps and statistics.

## Results

**Fig 3:**
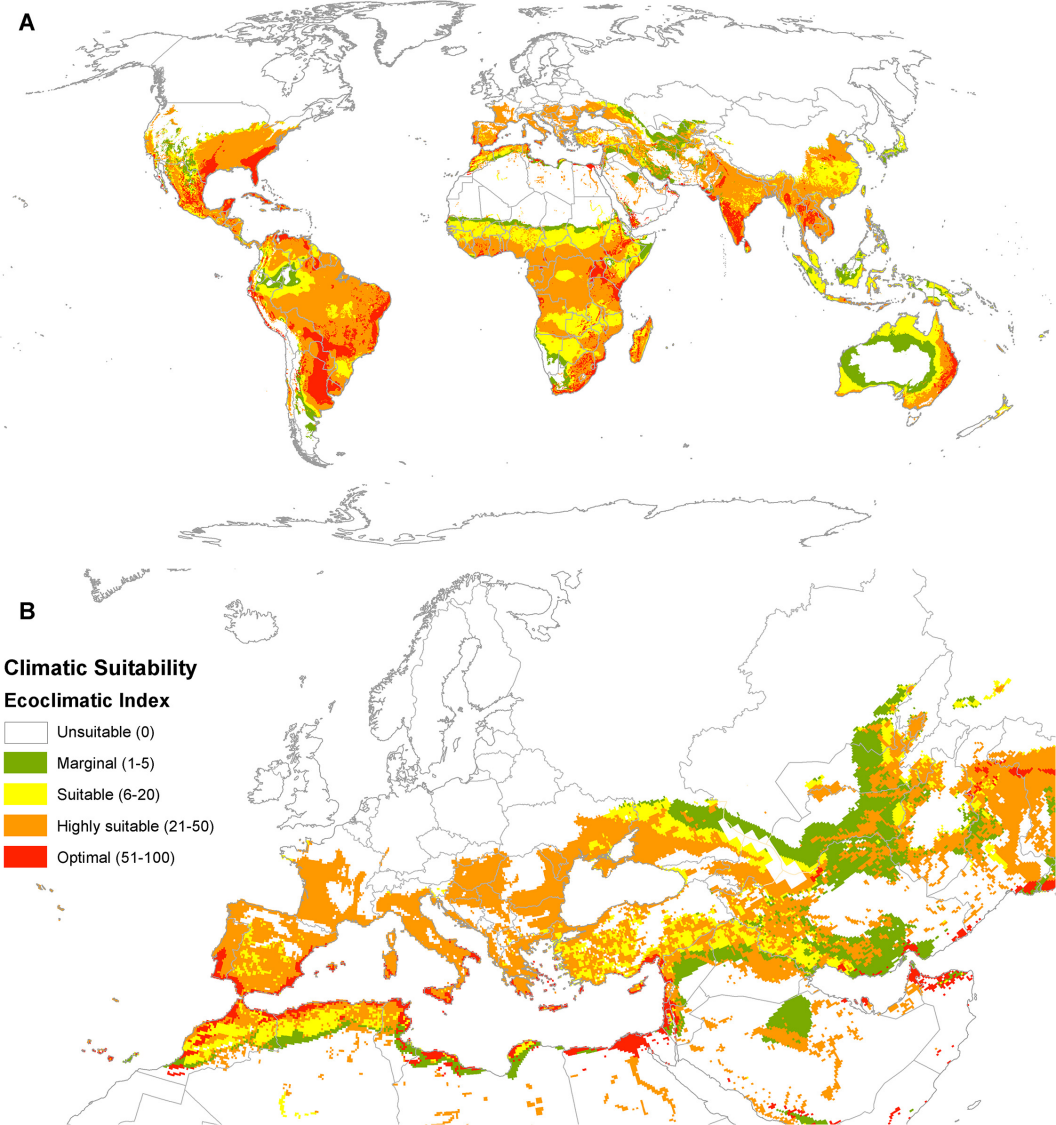
Climate suitability for *Parthenium hysterophorus* establishment modelled using CLIMEX with the CliMond CM10_1975H_WO_V1.1 climate dataset [9], including the effect of irrigation [41]. (A) Global and (B) Europe and North Africa.

**Fig 4:**
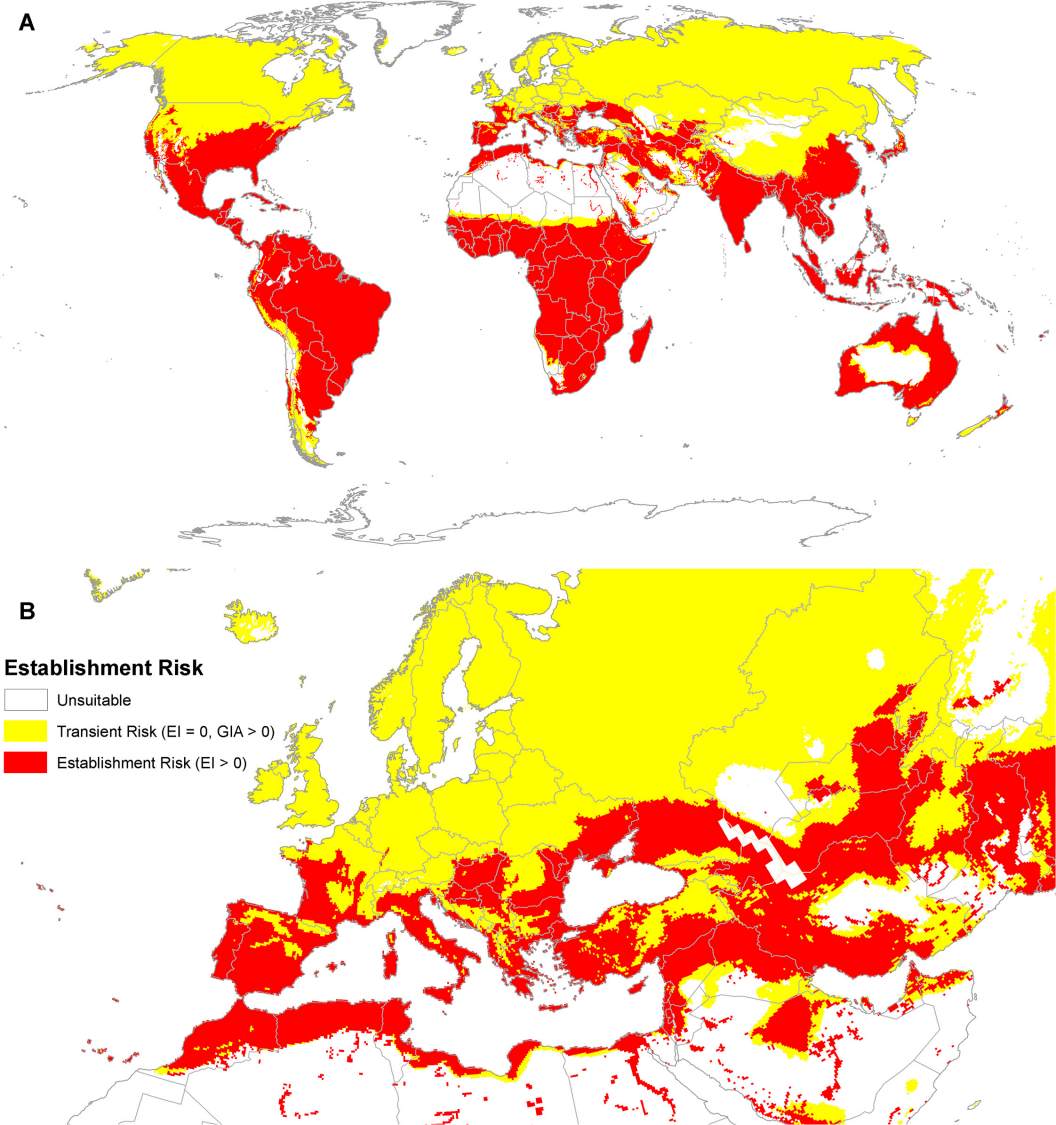
Combined establishment and transient invasion risks posed by *Parthenium hysterophorus* modelled using CLIMEX with the CliMond CM10_1975H_WO_V1.1 climate dataset [9], including the effect of irrigation [41]. (A) Global and (B) Europe and North Africa.

The modelled potential distribution of *P*. *hysterophorus* is very extensive, stretching from equatorial areas, through to warm temperate and Mediterranean climates (Fig 3). The effect of irrigation in extending the potential range into xeric regions is obvious in the scattered pockets of suitable locations in the western deserts of the USA (Fig 3A) and the Sahara Desert, where the Nile Valley is a particularly prominent feature (Fig 3B). The model also identifies that there is an additional, extremely large area in the northern hemisphere in which *P*. *hysterophorus* could pose a transient biosecurity risk (Fig 4). This accords with its observation in Belgium and Poland, where it was thought to be a transient. In its native range in the Americas, its modelled potential range extends into wet tropical areas, from which there are no recorded observations. Its modelled potential range for establishment in the USA is supported by a few northern location records. Extensive records in Asia in similarly cool conditions further support the conclusion that the plant can likely tolerate such cold conditions. In the wet tropics, consistent excessive soil moisture appears to prevent modelled population growth. In South America, the modelled potential range extends into colder regions than the recorded distribution (compare Figs 2 and 3).

In Eastern Asia and Australasia, the areas reserved for model validation, the model agreed perfectly with the known distribution (a model sensitivity score of 1.0). Model specificity was also good, with relatively few areas of range underlap. However, in China in particular, there appears to be considerable opportunity for in-filling invasion within the climatically suitable range.

**Fig 5:**
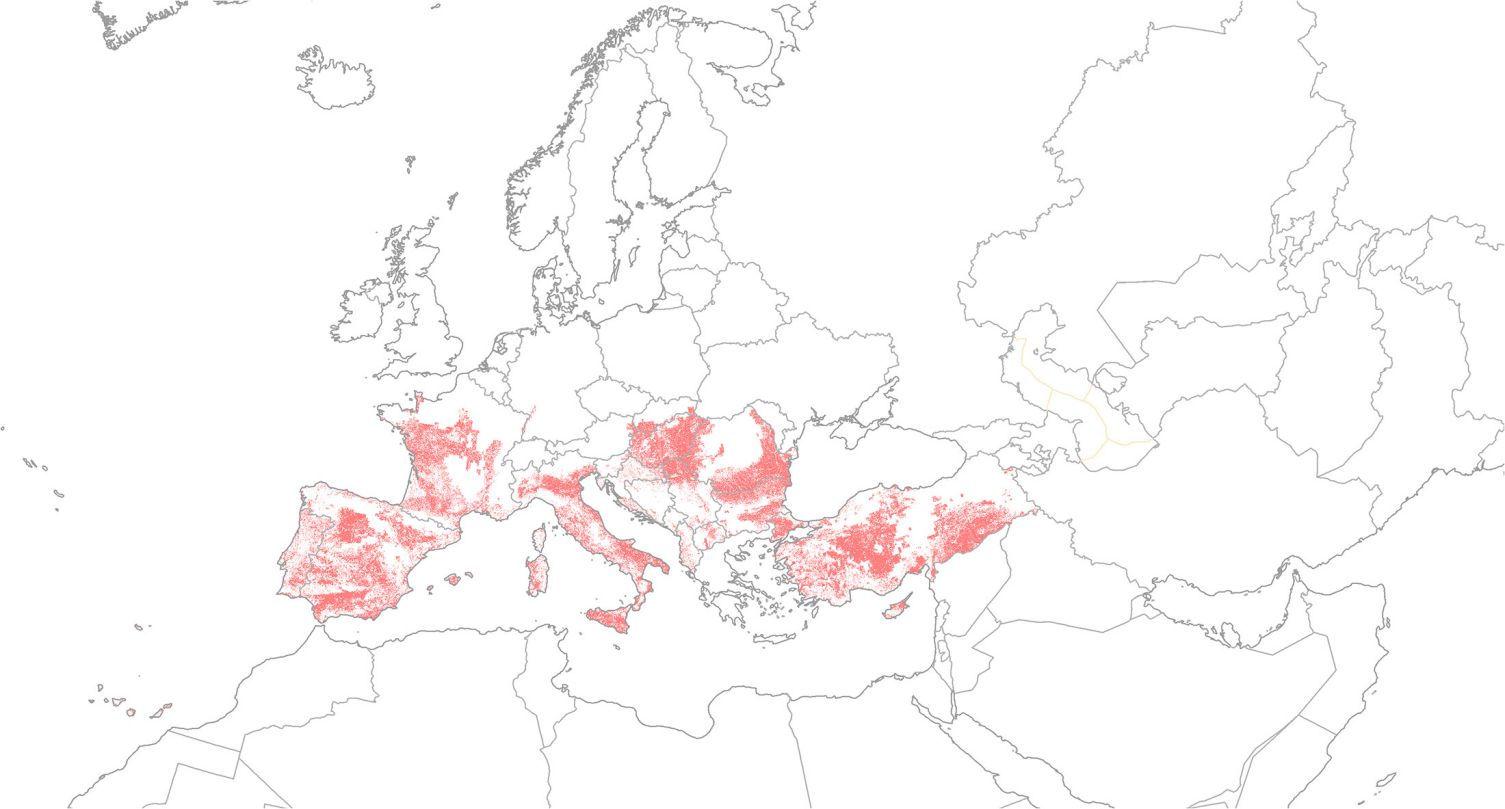
Endangered area considering climate (EI ≥ 1) and suitable habitat types in the CORINE database (http://www.eea.europa.eu/).

Within the EPPO region, the countries at risk are Albania, Algeria, Azerbaijan, Bosnia & Herzegovina, Bulgaria, Cyprus, Croatia, Former Republic of Macedonia, France, Greece, Hungary, Israel, Italy, Jordan, Kazakhstan, Kyrgyzstan, Malta, Moldova, Morocco, Portugal, Romania, Russia, Serbia, Slovakia, Slovenia, Spain, Tunisia, Turkey, Ukraine and Uzbekistan. The modelled climate suitability pattern is consistent with the reported transient nature of the plant populations in Belgium and Poland (Fig 4) [23,24]. Under the historical (current) climate scenario, more than 2 million ha of the EPPO region is apparently climatically suitable for establishment by *P*. *hysterophorus* (Table 2, Fig 5). Of this total area, less than half (approximately 946 000 ha) consists of habitat types considered suitable under the expert model (Table 2). The habitat classes considered at greatest risk (by area) are disturbed (urban, cropping and pastures). Perhaps also of cultural and economic significance is the threat to olive groves (100% of the plantations are at risk), vineyards (90%) and fruit and berry plantations (77%) may be threatened.

**Table 2:** Areal summary of composite invasion risk to Europe from *Parthenium hysterophorus* by habitat class according to the CORINE environmental database, considering climate with irrigation scenarios applied according to the GMIAV5 database [41]. Habitat classes are listed in descending order of area at risk under the current climate scenario. Land use is assumed to remain static under the future climate scenario.

				*Climate Scenario*					
*CORINE Code*	*CORINE Name*	*Suitable*	*Area (km* ^*2*^ *) Total*	*1975H*		*2080*		*Change in Area at risk (km* ^*2*^ *) EI ≥ 1*	*Percentage increase‡*
				*Area (km* ^*2*^ *) EI ≥ 1*	*Percentage of total area*	*Area (km* ^*2*^ *) EI ≥ 1*	*Percentage of total area*		
211	Non irrigated arable land	Y	1 212 530	536 661	44	1 029 382	85	492 721	92
321	Natural grasslands	Y	206 952	82 510	40	135 763	66	53 253	65
231	Pastures	Y	392 670	79 759	20	228 264	58	148 505	186
212	Permanently irrigated arable land	Y	81 519	71 185	87	80 877	99	9 692	14
333	Sparsely vegetated areas	Y	236 279	61 732	26	116 978	50	55 246	89
223	Olive groves	Y	37 560	37 445	100	37 557	100	112	0
221	Vineyards	Y	40 182	36 195	90	39 982	100	3 788	10
222	Fruit trees and berry plantations	Y	28 596	21 969	77	27 965	98	5 996	27
241	Annual crops associated with permanent crops	Y	9 458	9 281	98	9 439	100	158	2
511	Water courses	Y	13 115	6 283	48	9 758	74	3 474	55
133	Construction site	Y	1 862	1 258	68	1 634	88	375	30
122	Roads and rail networks and associated land	Y	2 546	1 037	41	2 130	84	1 093	105
141	Green urban areas	Y	3 046	688	23	2 159	71	1 471	214
132	Dump sites	Y	1 114	277	25	781	70	504	182
522	Estuaries	Y	540	149	28	295	55	147	99
000	Not classified		3 405 164	1 060 629	31	1 939 250	57	878 621	83
	**Total (suitable habitats only)**		**2 267 969**	**946 429**	**42**	**1 722 965**	**76**	**776 536**	**82**
** **	**Total (Climatically suitable)**	** **	**5 673 133**	**2 007 058**	**35**	**3 662 216**	**65**	**1 655 157**	**82**

^†^ The cells where the Ecoclimatic Index is positive, indicating potential for persistent populations to establish.

^***‡***^ Compared with the baseline area at risk under historical climate.

**Fig 6:**
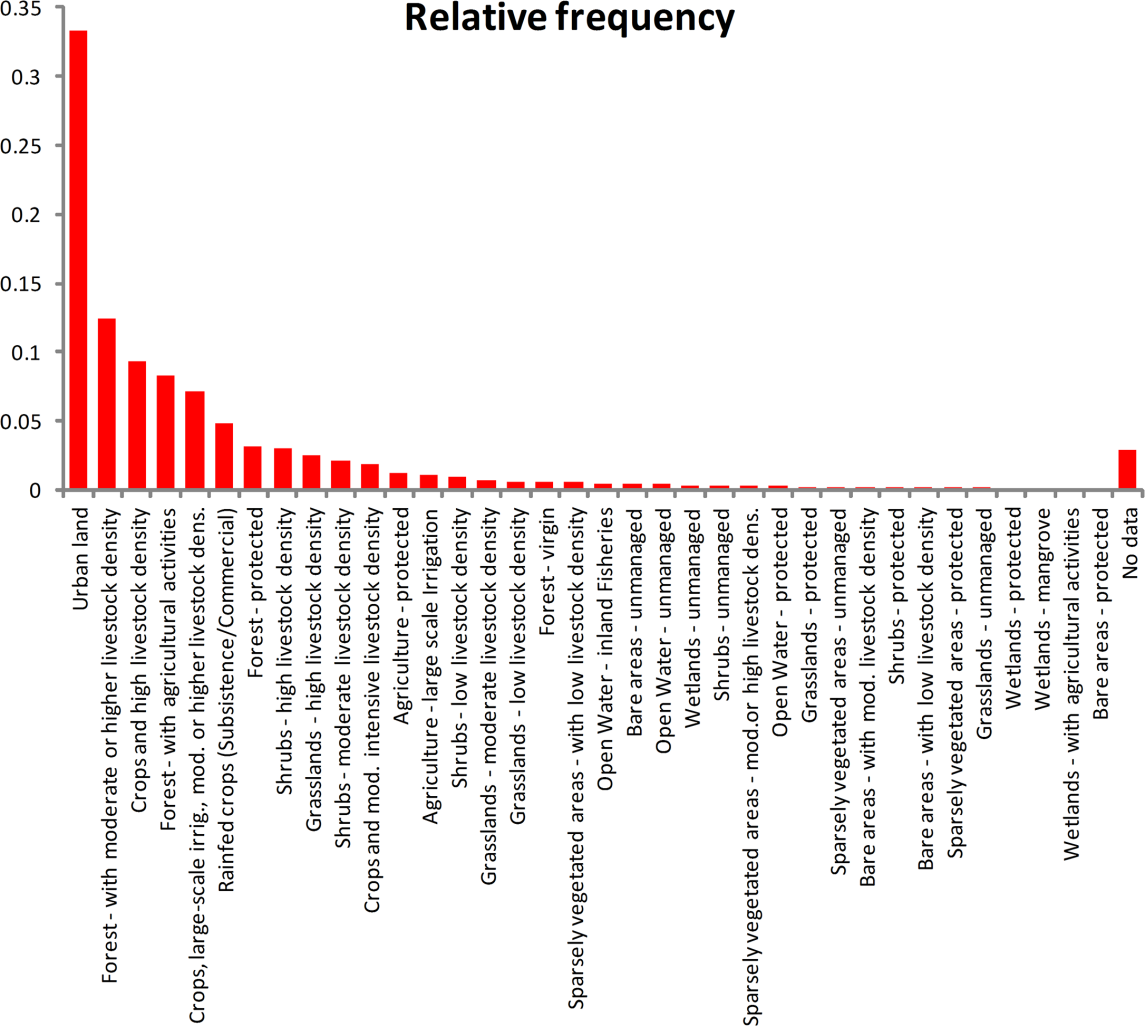
The relative frequency of land use systems in the FAO Land Use database overlain by location records for *Parthenium hysterophorus* from Fig 2.

Under the inferential FAO habitat model 29 land use classes were identified as being at risk in Europe, including cropping and pasture areas (Table 3, Fig 6). However, grazed forests and shrublands were also identified as being at risk (Table 3). The total area of suitable habitat in Europe modelled as at risk using the FAO dataset and the inferred habitat suitability classes was 1.6 million ha, nearly twice that from the CORINE dataset based on the expert opinion.

**Table 3:** Areal summary of composite invasion risk to Europe from *Parthenium hysterophorus* by land use system class according to the FAO Land Use Systems of the World database, considering climate with irrigation scenarios applied according to the GMIAV5 database [41]. Habitat classes are listed in descending order of area at risk under the historical (1975H) climate scenario.

				*Climate Scenario*					
*LUS Code*	*LUS Name*	*Suitable (expert assessment)^†^*	*Area (km* ^*2*^ *) TotalTotal*	*1975H*		*2080*		*Change in Area at risk (km* ^*2*^ *) EI ≥ 1*	*Percentage increase*
				*Area (km* ^*2*^ *) EI ≥ 1*	*Percentage of total area*	*Area (km* ^*2*^ *) EI ≥ 1*	*Percentage of total area*		
21	Crops and high livestock density	Y	767 150	269 283	35	683 276	89	413 993	154
04	Forest—with moderate or higher livestock density	Y	839 138	244 444	29	596 854	71	352 409	144
20	Crops and mod. intensive livestock density	Y	559 709	341 578	61	514 881	92	173 303	51
25	Urban land		614 847	262 436	43	460 600	75	198 164	76
19	Rainfed crops (Subsistence/Commercial)	Y	441 245	219 361	50	333 289	76	113 928	52
03	Forest—with agricultural activities		670 509	99 712	15	179 390	27	79 677	80
17	Shrubs—high livestock density	Y	202 972	88 824	44	167 145	82	78 321	88
22	Crops, large-scale irrig., mod. or higher livestock dens.	Y	146 219	123 945	85	140 798	96	16 853	14
11	Grasslands—high livestock density	Y	215 631	26 679	12	105 041	49	78 361	294
16	Shrubs—moderate livestock density	Y	101 199	77 713	77	90 508	89	12 795	16
33	Sparsely vegetated areas—mod.or high livestock dens.	Y	64 079	41 311	64	57 269	89	15 958	39
23	Agriculture—large scale Irrigation	Y	49 789	46 214	93	49 161	99	2 946	6
15	Shrubs—low livestock density	Y	57 545	39 910	69	46 321	80	6 411	16
02	Forest—protected		84 952	17 448	21	31 277	37	13 829	79
10	Grasslands—moderate livestock density	Y	40 424	13 345	33	30 915	76	17 570	132
40	Open Water—inland Fisheries		94 259	12 071	13	22 994	24	10 922	90
24	Agriculture—protected		34 909	14 304	41	22 892	66	8 588	60
13	Shrubs—unmanaged	Y	51 876	13 547	26	21 993	42	8 446	62
09	Grasslands—low livestock density	Y	20 584	3 382	16	10 081	49	6 700	198
37	Bare areas—with mod. livestock density		10 015	5 165	52	8 766	88	3 600	70
07	Grasslands—unmanaged	Y	64 781	2 573	4	8 459	13	5 886	229
30	Sparsely vegetated areas—unmanaged	Y	89 538	2 510	3	8 165	9	5 655	225
14	Shrubs—protected	Y	26 980	5 835	22	7 238	27	1 403	24
32	Sparsely vegetated areas—with low livestock density	Y	12 752	4 989	39	7 115	56	2 126	43
38	Open Water—unmanaged		16 296	2 875	18	6 519	40	3 644	127
34	Bare areas—unmanaged		55 631	1 549	3	4 990	9	3 442	222
39	Open Water—protected		8 078	2 394	30	3 887	48	1 493	62
27	Wetlands—protected		12 907	1 894	15	2 586	20	692	37
31	Sparsely vegetated areas—protected	Y	21 149	737	3	843	4	106	14
08	Grasslands—protected	Y	19 612	680	3	3 652	19	2 972	437
36	Bare areas—with low livestock density		3 946	351	9	577	15	227	65
35	Bare areas—protected		15 169	222	1	566	4	344	155
01	Forest—virgin		157 241	202	0	1 597	1	1 395	692
26	Wetlands—unmanaged		51 573	49	0	2 536	5	2 487	5048
28	Wetlands—mangrove		0	0	NA	0	NA	0	NA
29	Wetlands—with agricultural activities		0	0	NA	0	NA	0	NA
41	Undefined		0	0	NA	0	NA	0	NA
00	No data		48 054	18 888	39	28 768	60	9 880	52
	**Total (suitable habitats only)**		**3 792 371**	**1 566 862**	**41**	**2 883 005**	**76**	**1 316 143**	**84**
** **	**Total (Climatically suitable)**	** **	**5 670 756**	**2 006 422**	**35**	**3 660 948**	**65**	**1 654 526**	**82**

^†^ Considered equivalent to the classes identified as suitable using the expert assessment system (Table 2).

**Fig 7:**
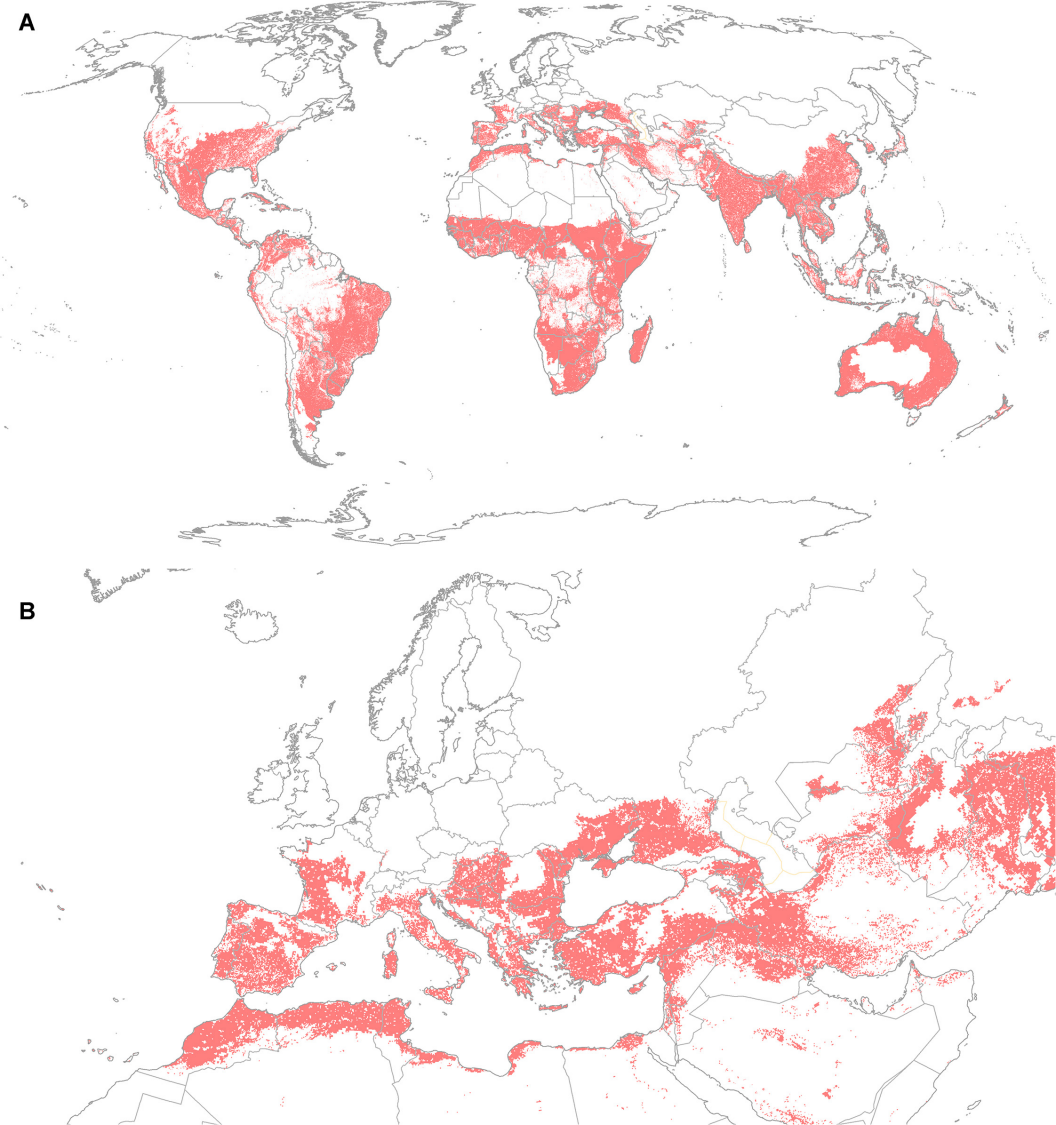
Endangered area considering climate (EI ≥ 1) and suitable habitat types in the FAO Land Use Systems database, A) Globally, and B) for Europe and North Africa.

The global risk patterns based on the inferential FAO model are similar to those for the expert-based system applied to Europe (Table 4, Fig 7B). However, there are some interesting differences: there was a significant number of records collected from areas classed as open water or wetlands. The likely causes are discussed below.

**Table 4:** A real summary of composite global invasion risk from *Parthenium hysterophorus* by land use system class according to the FAO Land Use Systems of the World database, considering climate with irrigation scenarios applied according to the GMIAV5 database [41]. Habitat classes are listed in descending order of area at risk under the current climate scenario.

				*Climate Scenario*					
*LUS Code*	*LUS Name*	*Suitable*	*Area (km* ^*2*^ *) Total*	*1975H*		*2080*		*Change in Area at risk (km* ^*2*^ *) EI ≥ 1*	*Percentage increase*
				*Area (km* ^*2*^ *) EI ≥ 1*	*Percentage of total area*	*Area (km* ^*2*^ *) EI ≥ 1*	*Percentage of total area*		
21	Crops and high livestock density	Y	9 097 883	7 125 110	78	8 355 326	92	1 230 216	17
04	Forest—with moderate or higher livestock density	Y	10 586 798	7 396 382	70	8 565 303	81	1 168 921	16
20	Crops and mod. intensive livestock density	Y	5 432 072	3 443 212	63	4 055 564	75	612 352	18
25	Urban land		3 426 546	2 449 779	71	2 938 205	86	488 425	20
19	Rainfed crops (Subsistence/Commercial)	Y	4 664 537	3 235 111	69	3 609 107	77	373 996	12
03	Forest—with agricultural activities		11 221 724	7 739 025	69	8 449 791	75	710 766	9
17	Shrubs—high livestock density	Y	2 534 303	2 227 217	88	2 412 489	95	185 272	8
22	Crops, large-scale irrig., mod. or higher livestock dens.	Y	2 533 662	2 257 272	89	2 274 656	90	17 383	1
11	Grasslands—high livestock density	Y	3 238 334	2 279 038	70	2 560 755	79	281 716	12
16	Shrubs—moderate livestock density	Y	3 524 259	2 934 208	83	3 261 094	93	326 886	11
33	Sparsely vegetated areas—mod.or high livestock dens.	Y	3 745 677	2 261 729	60	2 674 031	71	412 302	18
23	Agriculture—large scale Irrigation	Y	604 594	541 522	90	551 845	91	10 323	2
15	Shrubs—low livestock density	Y	3 307 702	2 115 093	64	2 330 736	70	215 643	10
02	Forest—protected		5 116 042	3 032 127	59	3 373 957	66	341 831	11
10	Grasslands—moderate livestock density	Y	3 244 887	2 057 197	63	2 427 023	75	369 826	18
40	Open Water—inland Fisheries		2 222 456	629 368	28	861 165	39	231 797	37
24	Agriculture—protected		763 630	575 494	75	607 549	80	32 055	6
13	Shrubs—unmanaged	Y	2 306 864	354 994	15	460 610	20	105 616	30
09	Grasslands—low livestock density	Y	2 892 336	1 211 031	42	1 399 012	48	187 981	16
37	Bare areas—with mod. livestock density		2 363 935	1 031 611	44	1 345 116	57	313 505	30
07	Grasslands—unmanaged	Y	1 818 515	281 373	15	339 896	19	58 523	21
30	Sparsely vegetated areas—unmanaged	Y	4 263 852	221 897	5	370 290	9	148 393	67
14	Shrubs—protected	Y	1 248 538	679 303	54	729 522	58	50 219	7
32	Sparsely vegetated areas—with low livestock density	Y	4 292 774	1 187 823	28	1 586 742	37	398 919	34
38	Open Water—unmanaged		309 754	110 208	36	133 015	43	22 807	21
34	Bare areas—unmanaged		12 841 091	624 247	5	1 260 891	10	636 644	102
39	Open Water—protected		371 179	81 246	22	100 596	27	19 350	24
27	Wetlands—protected		320 843	179 252	56	191 790	60	12 537	7
31	Sparsely vegetated areas—protected	Y	1 155 717	120 862	10	143 784	12	22 922	19
08	Grasslands—protected	Y	1 459 087	434 382	30	458 149	31	23 766	5
36	Bare areas—with low livestock density		4 716 441	449 284	10	1 016 832	22	567 549	126
35	Bare areas—protected		2 722 880	101 499	4	144 151	5	42 652	42
01	Forest—virgin		13 339 558	3 477 434	26	3 644 973	27	167 539	5
26	Wetlands—unmanaged		1 890 670	851 656	45	903 999	48	52 343	6
28	Wetlands—mangrove		62 640	57 520	NA	61 585	NA	4 066	NA
29	Wetlands—with agricultural activities		27 314	27 045	NA	27 314	NA	269	NA
41	Undefined		7 050	4 622	NA	4 869	NA	247	NA
00	No data		821 784	453 463	55	556 233	68	102 771	23
	**Total (suitable habitats only)**		**71 952 390**	**42 364 756**	**59**	**48 565 933**	**67**	**6 201 176**	**15**
** **	**Total (Climatically suitable)**	** **	**134 497 927**	**64 239 635**	**48**	**74 187 964**	**55**	**9 948 329**	**15**

^†^ Considered equivalent to the classes identified as suitable using the expert assessment system (Table 2).

### Climate change impacts on pest risk

**Fig 8:**
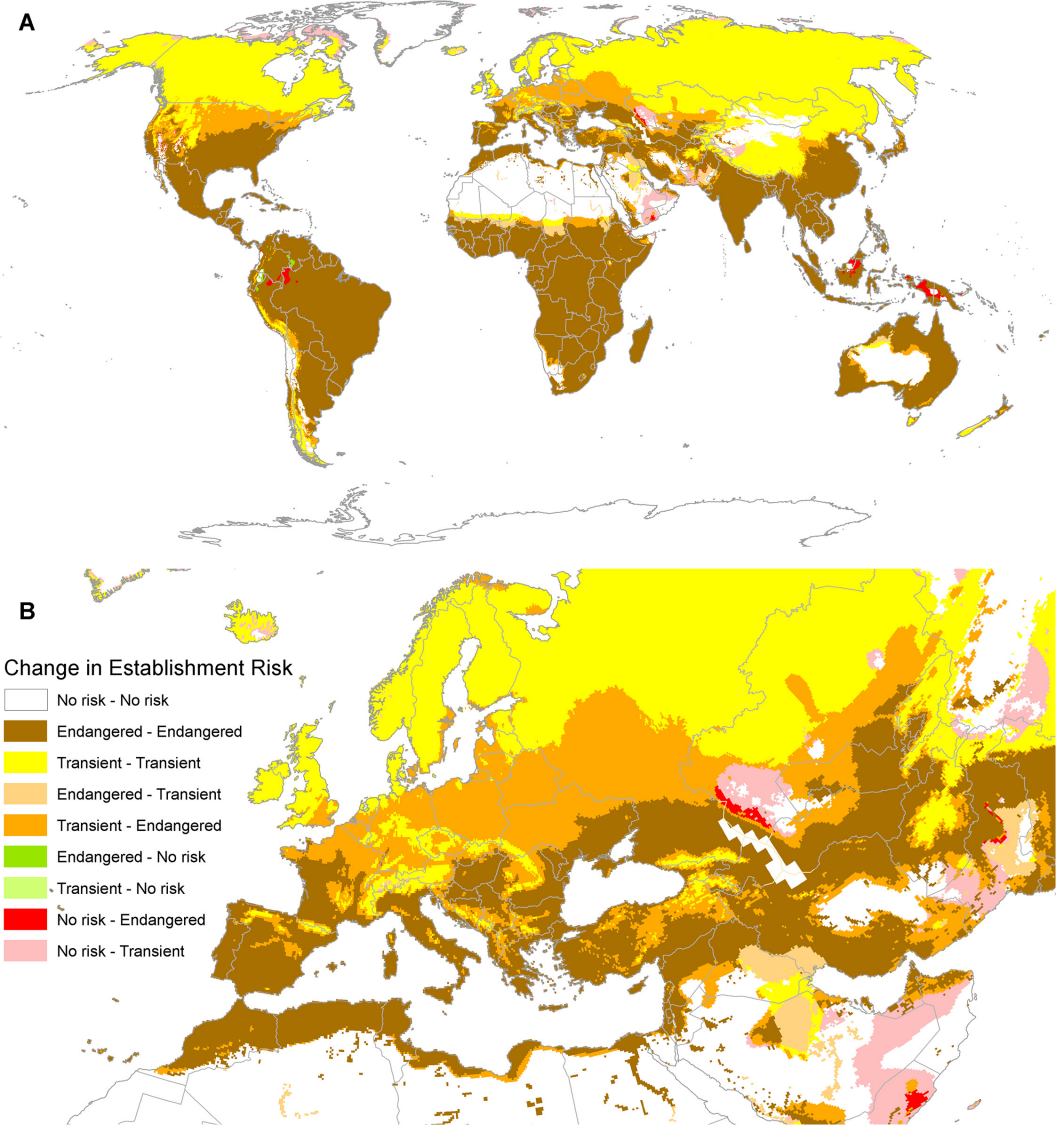
Change in climatic establishment risk for *Parthenium hysterophorus* comparing the CM10_1975H_V1.1 historical climatology and the CliMond. CM10_2070_CS_A2_V1.1 climate scenario. (A) Global and (B) Europe and North Africa.

Under the climate change scenario explored here, in the Northern Hemisphere, the modelled pest risks from *P*. *hysterophorus* extend further poleward compared with the current climate risks (Fig 8A, see Table 5 for legend description). The USA, continental Europe and northern Middle East are particularly sensitive to this scenario, with the risks changing from transient to endangered over huge areas. There is also a marked band along the equator where decreasing rainfall conditions could allow highland areas of western South America, Central Africa and South East Asia to become endangered by *P*. *hysterophorus* (Fig 8A).

**Table 5:** Summary of modelled pest risk change classes under the 2080 climate scenario.

Code	Current model	2080 projections	Is there a change?	Pest risk outcome	Colour used in mapping
1	No risk	No risk	No	Positive	white
2	Endangered	Endangered	No	Neutral	brown
3	Transient	Transient	No	Neutral	yellow
4	Endangered	Transient	Yes	Positive	50% orange
5	Transient	Endangered	Yes	Negative	100% orange
6	Endangered	No risk	Yes	Positive	100% green
7	Transient	No risk	Yes	Positive	50% green
8	No risk	Endangered	Yes	Negative	100% red
9	No risk	Transient	Yes	Negative	50% red

Within the EPPO region, many countries that appear presently to face only transient risks from *P*. *hysterophorus* may become endangered in the future, due primarily to rising temperatures (Austria, Belarus, Belgium, Czech Republic, Germany, Estonia, Latvia, Lithuania, the Netherlands, Poland, Slovenia, the United Kingdom, as well as larger parts of Bosnia and Herzegovina, Hungary, Kazakhstan, Moldova, Russia, Slovakia, Switzerland, Turkey, Ukraine, the southern coast of Sweden) (Fig 8B). The modelled change in climate suitability represents a near doubling of the endangered area (Fig 8B, Table 4).

## Discussion

Despite its extensive present known distribution (Fig 2), the modelled global potential distribution of *P*. *hysterophorus* greatly exceeds this, particularly in Africa, Asia, Australia, and Europe. Within its native range, the climate in the Amazon basin appears suitable for *P*. *hysterophorus*, but possibly only in the presence of frequent disturbance that reduces competition from other vegetation. If human disturbance patterns are extended into this region, we may find that *P*. *hysterophorus* also extends its range there.

Whilst *P*. *hysterophorus* is present in Israel within the EPPO region, it is thought to be absent from Europe *per se*. There is clearly an opportunity to prevent, or at least slow the spread of *P*. *hysterophorus* into Europe through vigilant phytosanitary measures. The requirement for free trade pathways between member states means that Israeli exports to Europe may pose a significant threat to the other EPPO member states, and special phytosanitary measures may be worth considering. The movement of people and material from Africa and the Middle East are also dispersal pathways that should be of concern to European biosecurity managers.

Within Africa, Asia and Australia, biosecurity measures to slow the spread of *P*. *hysterophorus* may still be worthwhile. Careful consideration of the present and potential distributions in these regions may assist with targeting education material and regulatory measures aimed at minimising impacts and reducing the rate of spread of this damaging invasive alien plant.

Extending the biological control programme against *P*. *hysterophorus* to Israel and other invaded countries is worthy of consideration. It may also be economically attractive for European states at risk of invasion by *P*. *hysterophorus* to co-invest in biological control measures in Israel and other places that pose a source threat.

### Habitat factors

Irrigation has an important effect on extending the range of *P*. *hysterophorus*, particularly in Saharan Africa, the Middle East and Central Australia. Conversely, within Europe, restricting the endangered area by using habitat types refines the area at risk considerably within the climatic range. These analytical elements could aid in refining economic impact analyses, and also perhaps in informing surveillance and rapid responses to incursion detections.

The spatial analysis of the distribution data for *P*. *hysterophorus* using the FAO dataset was revealing; expanding the range of habitat types beyond those identified by the expert assessment process. The association between the open water and wetland land use classes and *P*. *hysterophorus* was surprising given that *P*. *hysterophorus* does not grow in waterlogged situations. However, *P*. *hysterophorus* does grow on floodplains [56], so it is likely that the location records fall within riparian zones within the coarse open water and wetland land use classes. Similarly, during the expert deliberations, forested areas were discounted as suitable habitat on the grounds that *P*. *hysterophorus* reportedly grows poorly under shaded conditions, and would therefore be unable to persist. The FAO dataset comparison underscores the fact that forests (particularly those that are actively managed) are frequently a mosaic of different seral stages, and that ruderals such as *P*. *hysterophorus* can persist either through recolonisation or the maintenance of seed banks [57]. The more granular spatial resolution of the CORINE database is reflected in a larger set of habitat classes than the FAO dataset. Both of these factors make the CORINE database inherently less likely to create confusing interpretation problems with spatial intersections, as happened with the FAO dataset. However, the limitation usually lies in the spatial resolution of the location records for invasive alien species, rather than the habitat/land use data. This is especially marked for species location data collected prior to the widespread availability of GPS units. Hence, it is unclear whether chasing a finer-scale, globally-conformal, land use/habitat type classification would result in a more accurate assessment of the non-climatic habitat risk factors.

Whilst the fine spatial resolution of the CORINE database may be highly valued for risk assessment in the EPPO region, the lack of conformal global coverage is clearly a drawback for estimating non-climatic habitat risk factors for invasive alien species that have little or no history in the risk assessment area. The large size of the CORINE database also created practical challenges for spatial analyses in geographical information systems, sometimes requiring the dataset to be split in two for spatial intersections. One option for pest risk analysts is to sacrifice some precision for potentially greater accuracy, employing the FAO method and dataset as we have demonstrated here. Another option is to use a hybrid two-phase method combining the insights gained through the FAO dataset analysis with expert opinion to select classes from the CORINE database.

### Responding to climate change impacts on invasion risks

As the rate of change and the extent of future climatic changes are unknown (and largely unknowable), it is impossible and imprudent to use climate change scenarios such as the one presented here to inform future biosecurity policies and plans directly. Rather, the risk exposure revealed here should be used as the basis for understanding the nature of biosecurity decisions and their consequences under an inherently uncertain pattern of changing risks. In those areas where the future climate scenario risk maps indicate a risk of transient populations of *P*. *hysterophorus*, less effort may be placed on prevention, detection, and rapid response to this weed. However, if the risks might change in the future due to potential climate changes, several adaptation options present themselves (Table 6). It is imprudent to invest in expensive measures to address a problem that may not eventuate. The fact that the climate change scenario indicates that the risks for Europe are likely to increase in the future adds further weight to the conclusion that the present invasion risks by *P*. *hysterophorus*, based on historical climate, are significant. In the case of *P*. *hysterophorus* in the EPPO region, the climate change analysis adds little to the conclusion that there is a significant area at risk. The most cost-effective response may therefore be to consider what measures can be undertaken to stop the spread of *P*. *hysterophorus* out of Israel, or from other countries into the EPPO region, as well as to prevent its entry in EPPO countries at risk.

**Table 6:** Possible responses to potentially emerging pest risks under a rapidly changing climate.

*Response*	*Advantages*	*Disadvantages*	*Exemplar responses*
Prepare for the worst possible future risk case	Conservative approach, which may yield collateral protective benefits for measures that protect against multiple pests.	Immediate expenditure on protective measures against future risks that may not materialise	Implement measures to prevent the entry and spread of *P*. *hysterophorus*.
Ignore the emerging risks	No up-front expenditure due to emerging threats.	If emerging risks are realised, then unnecessary biosecurity failures may occur.	Maintain existing policies and practices; reacting to changing risks
Actively monitor changing risk patterns	Relatively small initial outlay on actively monitoring emerging risks. Little risk of over-investment.		Sentinel experiments, and active monitoring of changing risk patterns in analogue climates intermediate between those where it is presently capable of establishment, and those of the jurisdiction under consideration

### Model limitations

The CLIMEX model was fitted using the best available data and understanding available at the time of the analysis. However, we should be mindful that climate and distribution data are imperfect. The spatial resolution of the distribution data varied, and the estimated precision was not always reported. The mismatch between the resolution of the land use dataset and the species distribution data had the potential to pick up spurious habitat associations; hence we were careful to scrutinise low frequency associations. We should also be mindful that the CLIMEX Compare Locations model is a simplification of the complex ecological processes that define a species niche. The land use classification in the FAO dataset and the identification of the irrigated areas will doubtless contain minor spatial and classification errors. The mis-fitting points at the dry end of *P*. *hysterophorus’* range indicate a limit to the spatial precision in the global irrigated area database. However, despite these sources of potential errors, the analysis appears suitable for its intended purpose–to provide an indication of areas at risk of invasion should *P*. *hysterophorus* be introduced. Each of the mis-fitting points was in close spatial association with areas that were indicated as being suitable, and for which there were location records. This underscores the notion that the resulting maps should be used in aggregate to inform regional risk patterns, rather than being scrutinised at the level of an individual cell. In the extreme xeric and cold limits habitat suitability will be more subject to unusual micro-habitat variations that cannot be accounted for with global datasets and modelling.

With the climate change scenario it is important to remember that we are not applying observation data about the future. We have selected a single plausible scenario with which to stress-test the biosecurity conclusions of our niche modelling. Biosecurity managers should not make plans on the basis that the climate change scenario results presented here will eventuate. This could lead to an expensive waste of resources. Rather, managers should seek to understand firstly whether the scenario changes the invasion risks significantly within their jurisdiction. If so, they should consider what adaptive management processes they might prudently implement to monitor and manage that potential emerging threat, taking into account lead times for any adaptation measures.

### Advancing pest risk modelling

In this paper we applied two advances in pest risk modelling: spatially-explicit irrigation scenarios, and the inferential derivation of non-climatic habitat classes. Both methods are relatively easy to apply using a GIS with the freely available irrigation and land use datasets. The explicit irrigation scenario method allows the niche model to describe the species niche using biologically realistic parameters. In the absence of this method, the model would be unable to identify correctly the habitats at risk in xeric environments, either under-predicting (biologically realistic parameters), or over-predicting (using biologically unrealistic parameters that allow persistence in xeric environments).

The inferential method of identifying suitable land use classes can clearly provide a degree of rigour to the downscaling process. However, it does not abrogate the responsibility of the modeller or risk assessor to evaluate the resulting list of habitats critically and sceptically. Low frequency or unexpected habitat types should serve as a warning sign of a potential error. Whilst the impact of the downscaling process on the estimated endangered area is substantial, it may have minimal implications for analyses of the economic impacts of invasive alien species where the impacts apply to industries with well-defined spatially-explicit production characteristics. However, for species whose impacts are related to the area occupied, and affect natural environments, these downscaling methods could make a substantial difference to the results.

## Dedication

This paper is dedicated to the memory of Robert (Bob) Sutherst, who developed the CLIMEX modelling system, and who was a pioneer in the field of computer-based pest risk modelling. Sadly, Bob passed away the week before the work for this paper commenced.
